# Astragaloside IV inhibits microglia activation via glucocorticoid receptor mediated signaling pathway

**DOI:** 10.1038/srep19137

**Published:** 2016-01-11

**Authors:** Hong-Shuai Liu, Hai-Lian Shi, Fei Huang, Karin E. Peterson, Hui Wu, Yun-Yi Lan, Bei-Bei Zhang, Yi-Xin He, Tyson Woods, Min Du, Xiao-Jun Wu, Zheng-Tao Wang

**Affiliations:** 1 Shanghai Key Laboratory of Complex Prescription, The Ministry of Education (MOE) Key Laboratory for Standardization of Chinese Medicines, Institute of Chinese Materia Medica, Shanghai University of Traditional Chinese Medicine, Shanghai, 201203, China; 2 Laboratory of Persistent Viral Diseases, Rocky Mountain Laboratories, National Institute of Allergy and Infectious Disease, Hamilton, Montana, 59840, USA; 3 Unit of Immune Signaling and Regulation, Key Laboratory of Molecular Virology and Immunology, Institut Pasteur of Shanghai, Chinese Academy of Sciences, Shanghai, 200030, China

## Abstract

Inhibition of microglia activation may provide therapeutic treatment for many neurodegenerative diseases. Astragaloside IV (ASI) with anti-inflammatory properties has been tested as a therapeutic drug in clinical trials of China. However, the mechanism of ASI inhibiting neuroinflammation is unknown. In this study, we showed that ASI inhibited microglia activation both *in vivo* and *in vitro*. It could enhance glucocorticoid receptor (GR)-luciferase activity and facilitate GR nuclear translocation in microglial cells. Molecular docking and TR-FRET GR competitive binding experiments demonstrated that ASI could bind to GR in spite of relative low affinity. Meanwhile, ASI modulated GR-mediated signaling pathway, including dephosphorylation of PI3K, Akt, I κB and NF κB, therefore, decreased downstream production of proinflammatory mediators. Suppression of microglial BV-2 activation by ASI was abrogated by GR inhibitor, RU486 or GR siRNA. Similarly, RU486 counteracted the alleviative effect of ASI on microgliosis and neuronal injury *in vivo*. Our findings demonstrated that ASI inhibited microglia activation at least partially by activating the glucocorticoid pathway, suggesting its possible therapeutic potential for neuroinflammation in neurological diseases.

Microglial activation is one of the prominent characters of multiple sclerosis (MS), a progressive demyelinating disease of the central nervous system (CNS)^1^. In CNS, microglial cells have been regarded as resident innate immune cells and function as peripheral macrophages^2^ to defend against pathogens attack^3^. However, persistent and uncontrolled activation of microglia accumulates excessive detrimental substances, notably nitric oxide (NO), free radicals and proinflammatory cytokines (e.g. IL1β, TNFα), and finally lead to injury of neurons, particularly in MS^4^.

Glucocorticoids (GCs), a class of steroid hormones^5^,^6^, inhibit microglial activation^7^, through binding to glucocorticoid receptors (GRs)^8^. Upon ligand binding, GRs interact with signaling molecules in cytoplasm such as phosphoinositide 3-kinase (PI3K) or translocate into nucleus, where they repress the activity of other transcription factors (e.g. nuclear factor-κB, NF κB) or initiate gene transcriptions associated with anti-inflammatory effect^6^. Indeed, GCs trigger unsurpassed efficacy for treating MS^9^. Although used widely, chronic administration of GCs may result in severe complications like osteoporosis, obesity and diabetes, and increases the susceptibility to infectious diseases^10^. Therefore, different drugs with the same efficacy as GCs but lessened side effects will greatly benefit the clinical therapy of those inflammatory diseases.

Natural compounds from herbs are one of the important sources for development of new drugs. ASI is a cycloartane-type saponin molecule found in roots of *Astragalus membranes* with antiviral^11^,^12^, anti-hypertensive^13^, antioxidative^14^ and anti-inflammatory^15^ properties. Moreover, recent studies indicate that ASI can alleviate osteoporosis^16^, prevent diabetes and diabetic complications^17^,^18^,^19^,^20^,^21^ and reduce obesity (unpublished data). Clinical researches prove that ASI shows no adverse reaction and events^22^ and is safe and well tolerated^23^,^24^. And now the phase III clinical trial of ASI glucose on stable angina of coronary artery disease is ongoing in China. All of the studies suggested the efficacy and safety of ASI in the therapy of the mentioned diseases.

In our previous study, ASI was shown to inhibit microglia activation in experimental autoimmune encephalomyelitis (EAE) mice, an animal model for MS study^25^. However, its underlying molecular mechanism is still unclear. Since many natural saponin molecules such as ginsenoside-Rg1^26^, ginsenoside-Re^27^, protopanaxadiol and protopanaxatriol^28^ exert glucocorticord-like effect through GR, based on the molecule structure, we postulated that ASI probably also functions through GR as an active ligand. To testify the hypothesis, in current study, we examined the effect of ASI on suppression of microglial activation both *in vitro* and *in vivo*. Consequently, the effect of ASI on GR of microglia as well as its downstream signaling cascade was investigated. Our results indicated that ASI may be a safe and effective drug candidate for the therapy of neuroinflammation disorders characterized with microglial activation.

## Results

### ASI suppressed LPS-induced activation of BV-2 microglia cells

**Figure 1:**
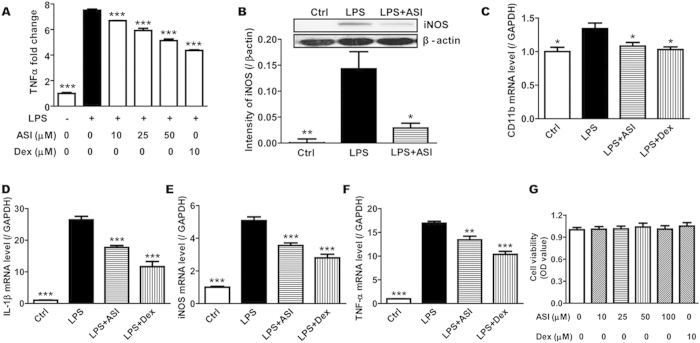
ASI inhibited the release of inflammatory mediators from BV-2 cells upon LPS stimulation. (**A**) ASI prevented release of TNFα measured by ELISA. (**B**) ASI inhibited iNOS protein expression. Inset showed the western blotting result of ASI on iNOS production. (**C–E**) ASI suppressed mRNA expressions of CD11b, IL1β, iNOS and TNFα. (**F**) ASI did not affect cell viability of BV-2cells. One-way ANOVA with Dunnett’s multiple comparison test was used for the statistics. *p < 0.05; **p < 0.01; ***p < 0.001 *vs* LPS group, N = 4/group, mean ± S.E.M. The data shown were one of the representative results from at least three independent experiments.

Stimulation of toll-like receptors (TLRs) results in potent activation of microglial cells. In particular, treatment of BV-2 microglial cells with the TLR4 ligand, LPS, significantly induced the upregulation of protein levels of proinflammatory factors TNFα (Fig. 1A, *p* < 0.001) and iNOS (Fig. 1B, *p* < 0.01) as well as mRNA levels of CD11b (Fig. 1C, *p* < 0.05), IL1β, TNFα and iNOS (Fig. 1D–F, *p* < 0.001). When treated with Dex (10 μM), the positive control drug, all of the proinflammatory factors were suppressed at both protein and mRNA levels. By contrast, ASI dose-dependently inhibited the release of TNFα from activated BV-2 cells (Fig. 1A, *p* < 0.001). Since ASI at 50 μM showed the best inhibitory effect, the dose was chosen for the subsequent experiments. Similar to Dex, ASI treatment (50 μM) also reduced the up-regulation of other proinflammatory factors at either protein or mRNA levels (Fig. 1B–F). To examine the effect of ASI and Dex on cell viability, CCK-8 method was used. As revealed in Fig. 1G, ASI at different doses (10, 25, 50 and 100 μM) did not influence cell viability of BV-2 cells. These results demonstrated that ASI reduced the inflammatory response of microglia upon stimulation with LPS.

**Figure 2:**
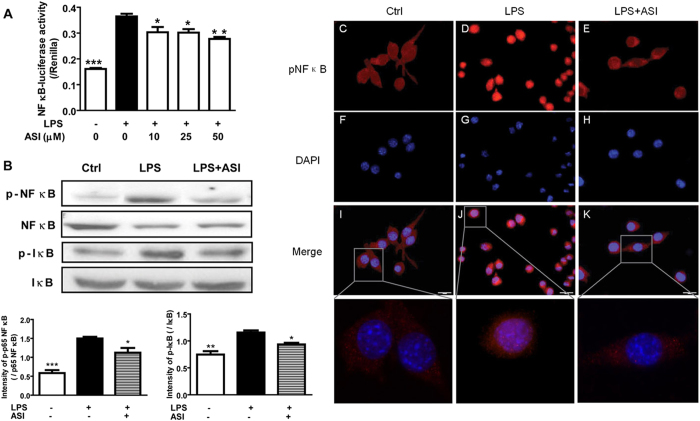
ASI prevented NF κB activation in LPS stimulated BV-2 cells. (**A**) ASI inhibited NF κB-luciferase activity after LPS stimulation. One-way ANOVA with Dunnett’s multiple comparison test was used for the statistics. *p < 0.05; **p < 0.01; ***p < 0.001 *vs* LPS group, N = 4/group, mean ± S.E.M. (**B**) ASI reduced phosphorylation of NF κB and I κB. (**C–K**) ASI prevented nuclear translocation of phosphorylated NF κB. Scale bar, 20 μm. The data shown were one of the representative results from at least three independent experiments.

To further confirm the inhibitory effect of ASI on microglia activation, BV-2 cells were analyzed for NF κB expression and localization. LPS (200 ng/ml) stimulation for 24 h led to dramatically increased NF κB-luciferase activity in BV-2 cells (Fig. 2A, *p* < 0.001). ASI at concentrations between 10 to 50 μM significantly reduced LPS-induced NF κB-luciferase activity (Fig. 2A, *p* < 0.05 or *p* < 0.01). Furthermore, results from western blotting assay showed that, ASI treatment could significantly reduce the upregulated phosphorylation of p65 NF κB and I κB stimulated by LPS in BV-2 cells (Fig. 2B). This suppression was confirmed by immunocytochemistry (ICC) analysis. In ICC analysis, ASI also inhibited the LPS-induced nuclear translocation of phosphorylated NF κB (Fig. 2C–K). Thus, ASI suppressed LPS-induced microglia activation by reduction of NF κB activation.

### ASI suppressed microglia activation and inflammatory responses in EAE mice

**Figure 3:**
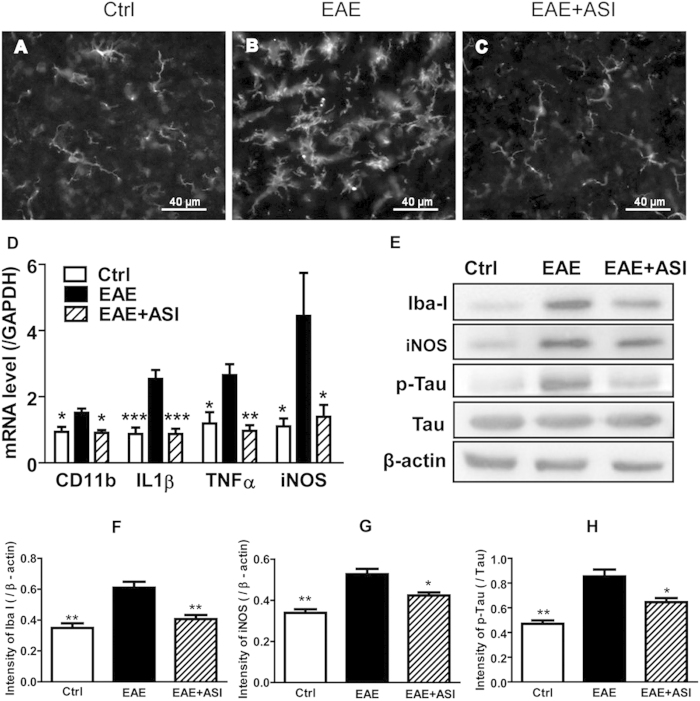
ASI administration deactivated microglial activation and reduced inflammatory responses in spinal cords of EAE mice. (**A–C**) Immunostaining results showed that ASI treatment decreased IbaI positive microglial activation in spinal cord of EAE mice. (**D**) ASI inhibited mRNA expressions of CD11b, IL1β, TNFα and iNOS. N = 4/group. (**E–H**) ASI suppressed protein expression of Iba-I and iNOS as well as the phosphorylation of Tau. One-way ANOVA with Dunnett’s multiple comparison test was used for the statistics. *p < 0.05; **p < 0.01; ***p < 0.001 *vs* EAE group, N = 3/group, mean ± S.E.M. The data shown were one of the representative results from at least three independent experiments.

To examine whether ASI had a similar effect on microglia cells *in vivo*, we analyzed microglia activation following EAE. Our previous study demonstrated that treatment with ASI reduced the severity of EAE as well as hippocampal microglia activation, however, the mechanism for this response was unknown^25^. Microglia activation is notable in neurological disease by a change in conformation and reactivity to the microglia marker, IbaI. In normal spinal cord of mice, fine processes of microglial cells were spread out (Fig. 3A). By contrast, in spinal cord of EAE mice, microglial cells were intensified with retracted processes and their numbers were increased (Fig. 3B). However, in ASI treated mice, the shape and density of microglia resumed to normal as that in physiological condition (Fig. 3C). Consistent with the IHC results, CD11b mRNA was slightly elevated in EAE mice (Fig. 3D), which was accompanied with significant up-regulation of proinflammatory markers IL1β, TNFα and iNOS mRNA (Fig. 3D). Protein levels of IbaI and iNOS were also upregulated (Fig. 3E–G) as well as phosphorylated tau protein (Fig. 3H), a marker of neuronal damage. Similar to the IHC results, the increased mRNA expression of CD11b, IL1β, TNFα and iNOS was also suppressed by ASI treatment (Fig. 3D). Furthermore, the increase in IbaI and iNOS protein levels as well as phosphorylation of tau was significantly attenuated in comparison with EAE mice without any treatment (Fig. 3E–H). These data demonstrated a remarkable reduction in microglial activation by ASI treatment, suggesting that ASI might be an effective therapy for neurodegenerative diseases with microglia activation.

### ASI GR-dependently decreased microglia activation

**Figure 4:**
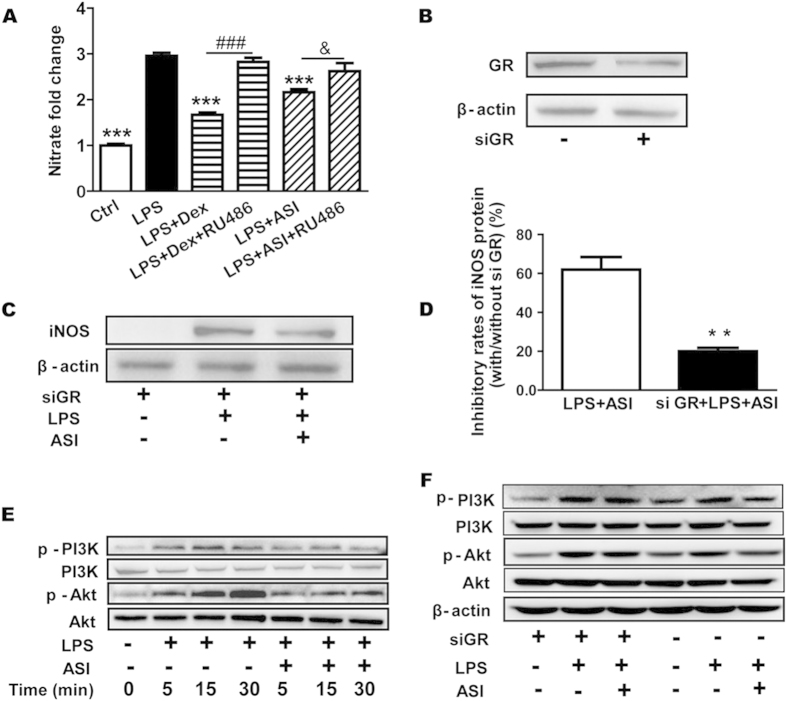
ASI decreased microglia activation depending on GR. (**A**) ASI inhibited NO production in BV-2 cells stimulated with LPS, the effect of which could be blocked by RU486. N = 4/group. One-way ANOVA with Dunnett’s multiple comparison test was used for the statistics. ***p < 0.001 *vs* LPS group; ^&^*p* < 0.05 *vs* LPS + ASI group; ^###^*p* < 0.001 *vs* LPS + Dex group, Dex, dexamethasone. (**B**) GR siRNA could effectively inhibit its protein expression. (**C**) Reduction of GR by siRNA decreased the inhibitory effect of ASI on protein expression of iNOS in BV-2 cells upon LPS stimulation. (**D**) Inhibitory rate of ASI on iNOS protein expression was significantly lower in siGR transfected plus LPS-stimulated BV-2 cells than that in LPS-stimulated BV-2 cells. Un-paired *t*-test was used for the statistics. **p < 0.01 *vs* LPS + ASI group, N = 3/group, mean ± S.E.M. (**E**) ASI time-dependently abolished phosphorylation of PI3K and Akt in BV-2 cells upon LPS stimulation. (**F**) si GR abolished ASI mediated downregulation of phosphorylation of PI3K and Akt in LPS-stumulated BV-2 cells. The data shown were one of the representative results from at least three independent experiments.

The mechanism by which ASI decreased microglia activation is not known. Since ASI is a natural saponin molecule and since other saponin molecules have been shown to have glucocorticoid-like effects, we examined whether ASI suppressed microglia activation through the GR. To determine if the anti-inflammatory effect of ASI was mediated through GR, we utilized RU486 (identical to mifepristone), an inhibitor of GR. Similar to previous results, LPS stimulation resulted in significantly increased release of NO, which could be attenuated by ASI (50 μM) treatment (Fig. 4A). Similar inhibition was observed with the known GR stimulator, Dex. RU486 reversed the inhibitory effects of both ASI and Dex on NO production (Fig. 4A), suggesting that ASI inhibited NO production through a GR-dependent manner.

To verify the role of GR in the inhibitory effect of ASI on microglia, siRNA of GR was used. As shown in Fig. 4B, GR siRNA could significantly down-regulate the expression of GR protein in BV-2 cells. LPS stimulation still induced iNOS expression in the GR siRNA-transfected cells (Fig. 4C). However, in these cells, the inhibitory efficacy of ASI on LPS-induced iNOS was significantly reduced when compared with the cells without transfection of GR siRNA (Fig. 4D).

As deactivation of PI3K or Akt is one of the properties of activated GR, we next examined whether ASI affected the phosphorylation of PI3K and Akt after LPS stimulation in BV-2 cells. As shown in Fig. 4E, LPS stimulation time-dependently induced phosphorylation of both PI3K and Akt. However, ASI treatment attenuated the activation of PI3K and Akt in a time-dependent manner, suggesting that ASI induced GR activation. To confirm the role of GR in the inhibitory effect of ASI on PI3K and Akt activation, the phosphorylation of PI3K and Akt with or without siGR was detected via western blotting assay. As displayed in Fig. 4F, the inhibitory effect of ASI on the phosphorylation of PI3K and Akt was abolished by siGR, suggesting that ASI modulated PI3K/Akt signaling via GR.

The combined data of inhibitor studies, pathway activation and siRNA treatment provided strong evidences that inhibitory effect of ASI on microglia activation was mediated through GR.

**Figure 5:**
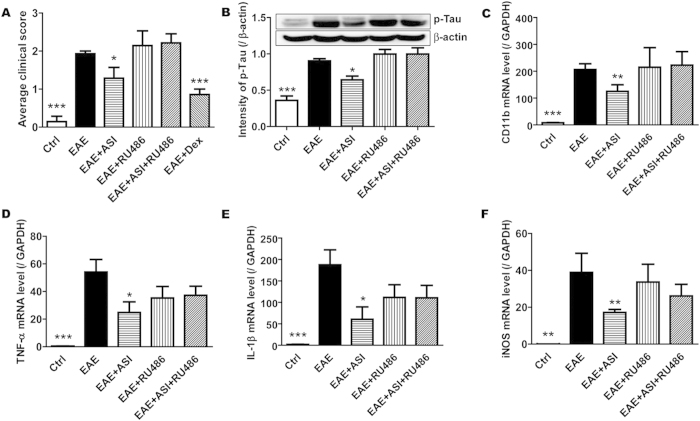
RU486 abrogated the inhibitory effect of ASI on microglial activation in EAE mice. (**A**) RU486 conteracted the inhibitory effect of ASI on clinical scores in EAE mice. (**B**) RU486 mitigated the inhibition of phosphorylation of Tau by ASI in spinal cords. (**C–F**) RU486 abolished the suppressive effect of ASI on gene expressions of CD11b, IL1β, TNFα and iNOS in EAE mice. One-way ANOVA with Dunnett’s multiple comparison test was used for the statistics. *p < 0.05, **p < 0.01, ***p < 0.001 *vs* EAE group, N = 7/group, mean ± S.E.M.

To determine if ASI also functioned through GR, *in vivo*, EAE mice were treated with or without ASI, RU486, ASI plus RU486, and Dex. As reported previously, ASI administration ameliorated the behavioral severity of EAE mice (Fig. 5A) and suppressed inflammatory responses associated with microglial activation. When co-treated with RU486, ASI could not improve EAE anymore as more phosphorylated tau proteins were found in spinal cords (Fig. 5B). Accordingly, ASI could not inhibit mRNA expressions of CD11b, TNFα, IL1β and iNOS in EAE mice as well (Fig. 5C–F). These data implicated the inhibitory effect of ASI on microglial activation in EAE mice was at least partly mediated by GR.

### ASI interacted with GR to decrease microglia activation

**Figure 6:**
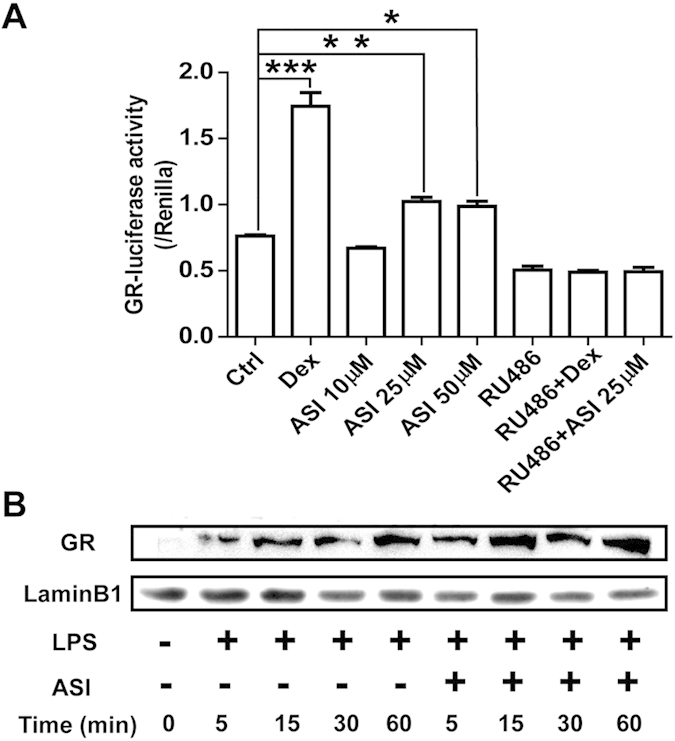
ASI enhanced GR activity in cells. (**A**) ASI increased GR-luciferase activity in HEK293 cells transfected with GR, the effect of which could be blocked by RU486. One-way ANOVA with Dunnett’s multiple comparison test was used for the statistics. *p < 0.05; **p < 0.01; ***p < 0.001 vs control group, N = 4/group, mean ± S.E.M. (**B**) ASI enhanced nuclear translocation of GR in BV-2 cells upon LPS stimulation. The data shown were one of the representative results from at least three independent experiments.

We next determined whether ASI affected GR activity. In HEK293 cells co-transfected with GR and GRE-luciferase, Dex induced high level of GR activity as measured by luciferase production (Fig. 6A). ASI treatment (25 or 50 μM) induced GR-dependent luciferase activity. Treatment of these cells with RU486 abolished both Dex- and ASI-induced luciferase activities, indicating the specificity of luciferase activity to GR stimulation.

To determine if ASI was actively involved in the translocation of GR, nuclear extracts of LPS-stimulated BV-2 cells with or without ASI treatment were analyzed by western blot. As shown in Fig. 6B, ASI promoted the translocation of GR into the nucleus of BV-2 cells at a much faster rate than cells stimulated with LPS alone. Thus, ASI appears to interact with GR and be involved in the translocation of GR to the nucleus of cells.

**Figure 7:**
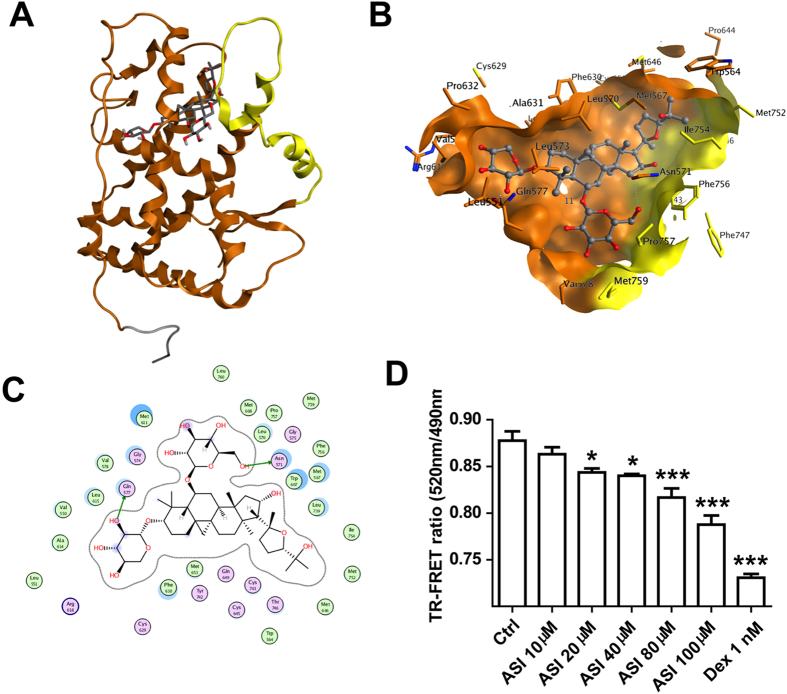
Refined docking poses of ASI into mGR SBD and TR-FRET GR competitive binding assay. (**A**) Overall structure of mGR and ASI complex. The gate region is highlighted in yellow. ASI was rendered in stick mode and colored by element: grey, carbon; red, oxygen. (**B**) View of binding pocket. ASI was rendered in ball-stick mode and colored by element. The protein residues were rendered in stick mode and the carbon was colored in residue color: brown, stable region; yellow, gate region. (**C**) 2D ligand-receptor interaction diagram. ASI had hydrophobic interaction with Met567, Leu570, Phe630, Met608, Met611, Ala612, Leu615, Met653, and Leu739. Two weak hydrogen bonds have been formed: one is between glucopyranosyl and the OE of Gln577, and another is between OH of glycoside and OD of Asn571. (**D**) ASI could dose-dependently bind to GR in TR-FRET GR competitive binding assay.One-way ANOVA with Dunnett’s multiple comparison test was used for the statistics.*p < 0.05; ***p < 0.001 vs control group, N = 4/group, mean ± S.E.M.

We next examined if ASI could directly bind to GR using a molecular docking experiment. An induce-fit docking was carried out to dock ASI into the ligand binding site of mGR. The initial docking complex of mGR and ASI had some atom clashes, therefore, a 10 ns MD simulation was tried to relax the system and remove clashes. The final complex showed that ASI could enter the “triangle” binding site (Fig. 7) that composed of many hydrophobic residues including Met567, Leu570, Met608, Met611, Ala612, Leu615, Phe630, Met653, and Leu739. In addition, two weak hydrogen bond could be formed: one was between glucopyranosyl and the OE of Gln577, and another was between OH of glycoside and OD of Asn571 (Fig. 7B,C). The hydrophobic interaction made major part of the ligand-receptor binding energy. Finally, to further confirm the result of molecular docking, a TR-FRET GR competitive assay was carried out. As shown in Fig. 7D, ASI could bind to human GR ligand binding domain (LBD) in a dose-dependent manner (0 to 100 μM).

**Figure 8:**
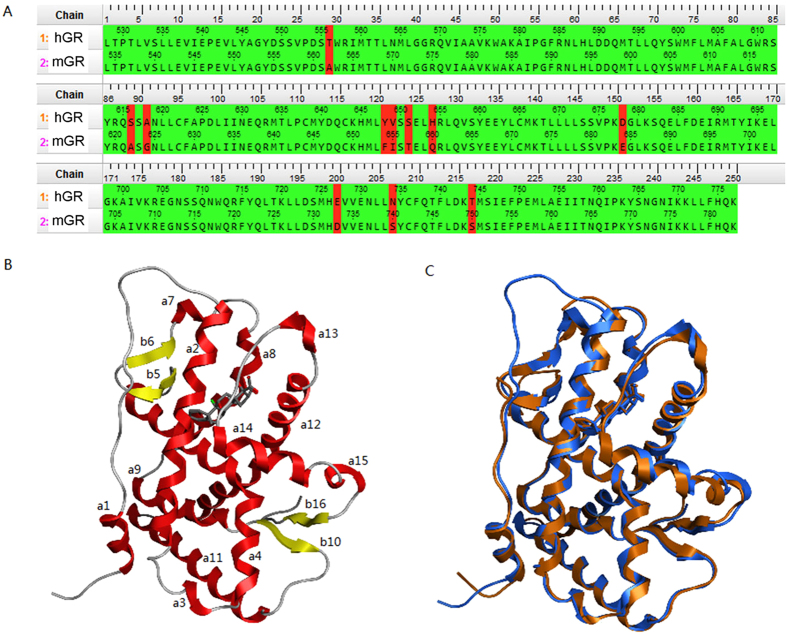
hGR and mGR’s SBDs are highly similar with each other in both sequence and 3D structure. (**A**) Sequence alignment of hGR and mGR SBD. (**B**) 3D structure (PDB ID: 1M2Z) of GR SBD. The different color represents different secondary structures: red, alpha-helix; yellow, beta-sheet; grey, random coil or turn. Generally, it is composed of 16 rigid components, out of which 12 are alpha-helix and 4 are anti-beta-sheet. a: alpha-helix; b: beta-sheet. The number after the letter stands for the order in sequence, from N-terminal to C-terminal. (**C**) The superposition of hGR SBD and mGR SBD complex: blue, hGR SBD (PDB ID: 1M2Z); grey, mGR SBD (PDB ID: 3MNE). Those two structures bind to the same ligand, dexamethasone, which has also been shown in the graph. Both the protein and ligand are superposed very well with each other, the RMSD of protein CA is 1.17 Å.

**Figure 9:**
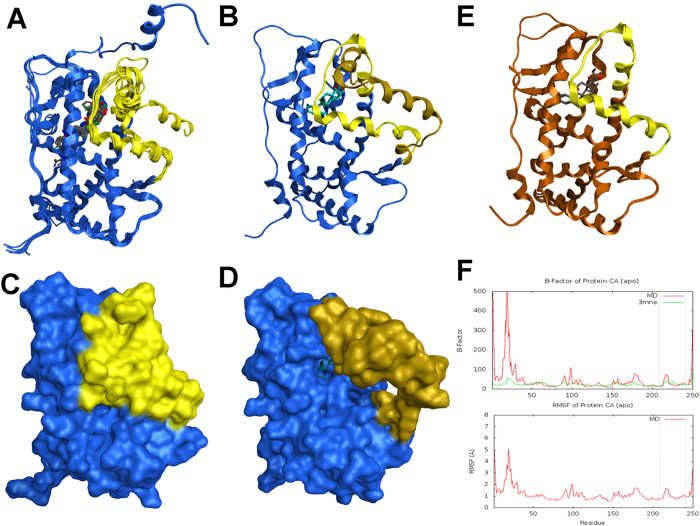
The structure of GR SBD is flexible and a “gate” may control the entry of ligand into the binding site. (**A**) Superposition of 9 hGR SBD (PDB ID: 1M2Z, 1NHZ, 1P93, 3BQD, 3CLD, 3ELC, 3H52, 3K22, and 3K23). The conformations are quite stable in most components except a1 and the gate region (Y735 to N768) highlighted in yellow. (**B**) The comparison between “gate close” and “gate open” conformations. The conserved structure is rendered in blue, the closed gate region is rendered in yellow and the opened gate region is rendered in deep yellow. (**C**) The surface view of “gate close” conformation. The ligand is deeply buried in protein. (**D**) The surface view of “gate open” conformation. The ligand can enter into the binding site through the open gate. (**E**) Superposition of 3 mGR SBD structures (PDB ID: 3MNE, 3MNO, and 3MNP). The gate region (Y741 to N774) is rendered in yellow. (**F**) The atom fluctuation plot of protein CA resulted from MD simulation of mGR “apo” structure from 3MNE. The top plot is the B-factor of CA: red, derived from 10 ns MD simulation; green, extracted from PDB file. The bottom plot is the RMSF (Root Mean Structure Fluctuation) of CA. The two dashed lines enclose residues contained in the gate region. The peak indicates that the linker between a13 and a14 are flexible and may cause movement of the “gate”.

To further examine the possible interactions of ASI with GR, we examined the binding domain of GR. The human glucocorticoid receptor (hGR) is highly similar with mGR in the steroid binding domain (SBD, Fig. 8A). They are composed of 250 aa, and the identity and similarity between them are 95.6% and 98.8%, respectively, with only 11 discrepant aa. The overall structure of GR SBD consists of 12 alpha helixes and 4 anti-parallel beta sheets (Fig. 8B) and is quite conserved between hGR and mGR with an average CA RMSD 1.17 Å (Fig. 8C). The SBD is surrounded by 7 alpha helixes and 2 anti-parallel beta sheets: a2, a4, b5, b6, a7, a8, a12, a13 and a14. However, the SBD appears to be very dense suggesting that a large ligand such as ASI may not be able to enter without any conformational change. This suggest that the SBD of GR may be flexible to allow a “gate” to be opened before the ligand arrives to allow binding. To test the hypothesis, multiple structures of hGR and mGR were collected to analyze the flexibility of the SBD in binding ligands. Nine structures of hGR SBD, which bound to variable size of ligands, were superposed (Fig. 9A). The superposed structures indicated that the N-terminal a1 and the long linker between a1 and a2 were very flexible. In 3H52, an hGR SBD-mifepristone complex, the a1 and the long linker after a1 projected to a vertical direction. However, they were still far from SBD. The a13, a14, and the linker between them were also very flexible. The structure that provided the most potential insight into binding was 1NHZ, also a hGR SBD-mifepristone complex, in which the a13 and a14 opened about 45° (Fig. 9B). Since a13 and a14 were close to SBD, the movement of a13 and a14 opened the binding site (Fig. 9C,D). These data suggested that the SBD of hGR could be opened at a13, a14 and linker, the “gate region”.

We also compared 3 structures of mGR SBD, which were all complexed with Dex (Fig. 9E). Most of the backbones were quite conserved except the linker between a1 and a2. From the limited mGR structures, a similar “gate open” conformation could not be observed. However, based on the observation of structural variability in hGR and also the highly sequence and structure homology between hGR SBD and mGR SBD, the data suggests that the “gate-like” mechanism might be also present in mGR to enhance its ability to fit big ligand like ASI. The MD simulation results of mGR SDB “apo” (without any ligand bound with protein in the X-Ray structure) state supported that the gate region (a13, a14 and the linker between them) of mGR SBD was also flexible (Fig. 9F). This indicated similar functions of ASI on GR in both humans and mice, suggesting that functional studies in mice might be highly relevant to human therapeutics.

## Discussion

**Figure 10:**
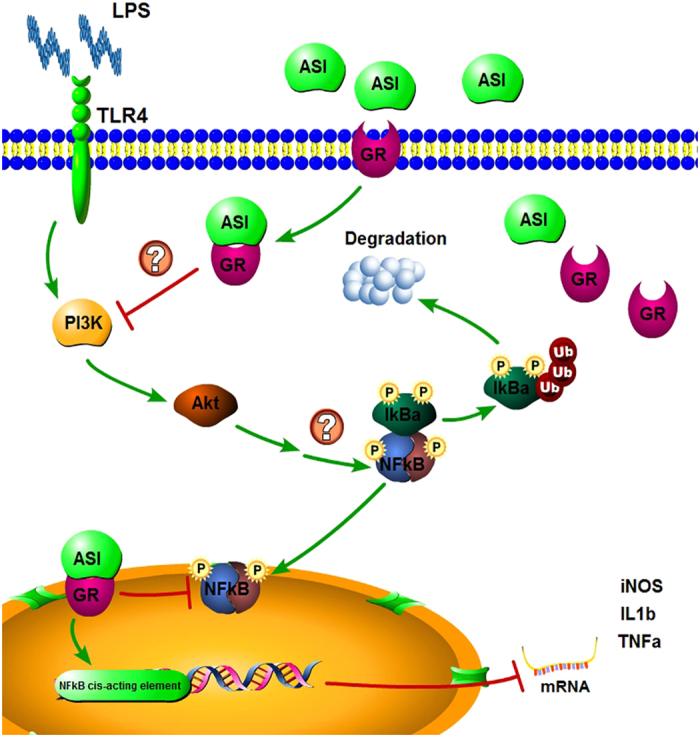
The schematic illustration of the molecular mechanism of ASI on GR signaling pathway. On the one hand, after binding to GR, ASI inhibited activation of PI3K and Akt, which reduced downstream phosphorylated I κB and led to the deactivation of NF κB. On the other hand, ASI and GR complex was translocated into nucleus, where it prevented NF κB to bind to its acting element and modulated downstream gene expressions of iNOS, TNFα and IL1β. The graph was made using Pathway Builder Tool 2.0 (Protein Lounge, San Diego, CA).

Total astragalosides and ASI have already been proved to be safe and well tolerated^24^. Moreover, ASI has already been approved as a novel drug for the phase I clinical study by the State Food and Drug Administration of China^23^, albeit for the treatment of cardiovascular diseases. Recent studies have indicated that ASI or total astragalosides may be therapeutic for neurological disease as shown by studies of traumatic brain injury in rats^29^ and EAE in mice^25^,^30^. However, the exact mechanism underlying or action pathway of ASI function had not been elucidated. In present study, we demonstrated that ASI inhibited microglia activation both *in vitro* and *in vivo*, via direct binding to GR, thereby, enhanced the nuclear translocation of GR and actively provoked the GR-mediated anti-inflammatory signaling pathways (Fig. 10), namely dephosphorylation of PI3K, Akt, IκB and NFκB, at last facilitated the control of downstream proinflammatory mediators.

Neuroinflammation shown as excessive microglial activation or microgliosis has been reported to be closely associated with many neurodegenerative diseases, including Alzheimer’s disease, Parkinson’s disease, and MS. Functional and lesional disturbances in CNS of those diseases often lead to further morphological changes of microglia with remarkable molecular signatures. Upon activation, microglial cells lose their normal ramified shape, retract processes and round up to the amoeboid morphology. In EAE mice, abundant activated microglia labelled with IbaI as well as elevated mRNA of CD11b, one of the integrin family members served as microglial marker, were found present in gray matter of spinal cord (Fig. 3). After ASI treatment, those microglia resumed to normal ramified shape, suggesting that ASI could decrease microglia activation, therefore, restrained further neuroinflammation.

Release of inflammatory mediators such as NO, IL1β and TNFα is one of the characteristics of overactivated microglia under inflammatory circumstances^31^. These cytotoxic factors can induce significant neurotoxic effects^32^. In brain, NO is produced by neuronal nitric oxide synthase (nNOS) and iNOS. However, microglia in normal physiological state do not express iNOS unless they are activated by various proinflammatory factors^33^,^34^. Not surprisingly, the elevated levels of iNOS, IL1β and TNFα are often regarded as hallmarks of neurodegenerative diseases^35^. In our study, significantly increased mRNA expressions of iNOS, IL1β and TNFα were found in spinal cords of EAE mice as well as that in BV-2 cells stimulated with LPS. However, ASI administration actively suppressed the elevation of those factors, showing strong anti-inflammatory effect. Accordingly, phosphorylation of tau, the indicator for neurodegeneration, was significantly reduced in EAE mice treated with ASI. Although multiple mechanisms might get involved in the alleviation of EAE, doubtlessly, suppression of microglia by ASI, therefore, preventing against further neurotoxin production played a crucial role in the process^25^.

NF κB is closely associated with chronic inflammatory diseases by driving the production of proinflammatory factors^36^,^37^. In the inactive state, NF κB dimers are binding to I κB proteins. Elicitors such as proinflammatory cytokines and LPS trigger the activation of the I κB kinase complex, resulting in consequential phosphorylation, ubiquitination, and degradation of I κB. The released NF κB dimmers will be translocated to the nucleus and initiate transcription of target genes via binding to specific DNA sequences. In our study, LPS stimulation enhanced phosphorylation of IκB, therefore, led to the activation of NF κB shown by elevated nuclear translocation of phosphorylated NF κB and transcription of downstream genes in BV-2 cells (Fig. 2). However, all these effects of LPS could be effectively abolished by ASI treatment, suggesting the involvement of NF κB pathway in the anti-inflammatory effect of ASI.

PI3K/Akt pathway is essential to LPS-induced microglial activation via its specific receptor (TLR4)^38^. And cross-talks have been implicated between NF κB and PI3K/Akt pathways^39^. Researches demonstrate that inhibition of PI3K/Akt efficiently abolishes NF κB mediated production of inflammatory cytokines in BV-2 cells upon LPS stimulation^40^,^41^. In our experiments, activation of PI3K and Akt induced by LPS could be abrogated by ASI. Since activation of GRs deactivates PI3K/Akt pathway^6^, and in our experiments, knock-down GR by siRNA counteracted the regulation of ASI on the phosphorylation of PI3K and Akt in BV-2 cells, it is reasonable to postulate that ASI prevented microglia activation via GRs. To corroborate our hypothesis, several other experiments were carried out. In HEK293 cells over-expressing GRs, ASI induced significant transactivation of luciferase that was controlled by GRE (glucocorticoid response element). Moreover, its effect could be blocked by RU486. In the *in vitro* competitive binding assay, ASI dose-dependently inhibited Fluormone labeled ligand binding to GRs. In the molecular docking assay, ASI was also found to dock into the ligand binding site of mGR. When GR was knocked down in BV-2 cells or blocked by RU486 in EAE mice, the anti-inflammatory effect of ASI was alleviated.

In short, all of these results strongly verified our hypothesis: ASI suppressed microglia activation via GRs to trigger its anti-neuroinflammatory effect. These novel findings may shed light on the development of ASI or its derivatives as an efficient and safe drug candidate in therapy of MS and other neurodegenerative diseases characterized by neuroinflammation.

## Materials and Methods

### EAE induction and treatment

Experimental autoimmune encephalomyelitis was induced in 6-week-old female C57BL/6 mice as described previously^42^. Each mouse was immunized subcutaneously with 300 μg of MOG_35–55_ that emulsified in complete Freund’s adjuvant (CFA) containing 400 μg of heat-inactivated *Mycobacterium tuberculosis* H37RA. The control mice were injected with adjuvant without MOG_35–55_. Intraperitoneal injection of pertussis toxin (200 ng/mouse) was given immediately and again 48 h later. Clinical behavior of mice was evaluated daily according to the criteria used by Peiris M and his colleagues^43^.

Administration of ASI (purity > 98%, 20 mg/kg) with or without RU486 (10 mg/kg) was intraperitoneally given daily one day before EAE induction and consecutively for 2 weeks. Three weeks later, the animals were subjected to either histopathology analysis according to the method described below or tissue collection after anesthetization with excessive 20% urethane.

All animal experiments were performed according to the protocols approved by Animal Care and Use Committee of Shanghai University of Traditional Chinese Medicine, which complies with international rules and policies. Ethics in accordance with the ARRIVE (Animal Research: Reporting *In Vivo* Experiments) guidelines were followed in the animal experiments and were approved by the Animal Care and Use Committee of Shanghai University of Traditional Chinese Medicine. All efforts were made to minimize suffering and reduce the number of animals used.

### Immunohistochemistry (IHC)

After anesthetization, mice were intracardially perfused with PBS followed by 4% paraformaldehyde in PBS. Coronal sections of spinal cords at 20 μm were obtained on a Leica 1950 cryostat. IHC procedure was performed according to the methods described previously^25^. Briefly, the sections were permeablized and blocked with 10% donkey serum in PBS containing 0.3% Triton X-100 for half an hour. Consequently, they were incubated with primary antibody against Iba I (Gene Tex, GTX100042) at 4 °C overnight followed by thoroughly wash with PBS. After incubation with secondary antibody conjugated with Alexa 488 (Invitrogen, A21208), the sections were mounted on slides. The fluorescence was visualized using an inverted fluorescent microscope (Olympus IX 81, Japan).

### BV-2 cell culture and treatment

Mouse microglial cell line obtained from American Type Culture Collection (ATCC) was maintained in DMEM medium supplemented with 10% fetal bovine serum at 37 °C with CO_2_. Cells were subcultured on 6-, 12- or 96-well plates at a density of 2 × 10^5^ cells/ml unless otherwise mentioned.

For mRNA expression pattern assay, cells were pre-treated with or without ASI (50 μM) or dexamethasone (Dex, 10 μM) for 2 h prior to LPS stimulation. After treated with LPS (200 ng/ml) for 24 h, cells were lysed in Trizol (Life Technologies). Total RNA was extracted according to the protocol provided by the manufacturer and stored in −80 °C thereafter.

For phosphorylated PI3K and Akt analysis, cells were pre-treated with or without ASI (50 μM) for 2 h followed by LPS (200 ng/ml) stimulation for 5, 15, 30 and 60 min. Thereafter, they were lysed with CelLytic^TM^ MT mammalian tissue lysis reagent (Sigma, USA) supplemented with protease inhibitor cocktail (Sigma, P3840) and phosphatase inhibitor cocktail 2 (Sigma, P5726) on ice for 0.5 h. The supernatant of the lysate after centrifugation at 12,000 rpm for 10 min at 4 °C was subjected to western blotting assay.

For the nuclear translocation assay of NF κB and GR, nuclear extract of cells was obtained with NE-PER® Nuclear and Cytoplasmic Extraction Reagents according to the manual of the manufacture (Thermo Scientific, USA), and thus subjected to western blotting analysis.

To evaluate the influence of GR knock-down on the anti-inflammatory effect of ASI, BV-2 cells were transiently transfected with GR siRNA (GCUGGAAGAAAUGAUUGCATT) or scrambled siRNA (UUCUCCGAACGUGUCACGUTT) using lipofectamine 3000 (Life Technologies). Twenty four hours later, the cells were pre-treated with or without ASI (50 μM) for 2 h followed by LPS (200 ng/ml) stimulation for another 20 h. Thereafter, the cells were harvested and lysed for western blotting analysis.

### Western blotting analysis

Proteins from cells or tissues were extracted by sonication in CelLytic^TM^ MT mammalian tissue lysis reagent with protease and phosphatase inhibitor cocktails. After centrifugation at 12,000 rpm for 10 min at 4 °C, the supernatant of the lysate was collected and the protein concentration was determined by BCA method. Totally, thirty microgram of proteins from each sample were separated by 10% SDS-PAGE and transferred onto PVDF membranes. After blocked with 5% skim milk, the membranes were incubated with respective primary antibodies against phospho-NF κB (p-Ser536, CST, 3033S), NF κB (CST, 6956), iNOS (Abcam, ab15323), phospho-I κB (p-Ser32, CST, 2859S), I κB (CST, 4812S), LaminB1 (Epitomics, 6581-1), GR (Eptomics, 3626-1), phospho-Tau (p-Ser202/Thr205, Pierce, MN1020), Tau (Abcam, ab3931), Akt (CST, 2920), p-Akt (p-S129, Epitomics, 550-1), p-PI3K (p-Tyr458, CST, 4228), PI3K (Epitomics, 3838-1) and β-actin (Sigma, A5441) and, sequentially, secondary antibodies conjugated with horseradish peroxidase (Life Technologies). The signal was visualized with the ECL prime kit (GE Healthcare, UK). Relative quantification of the bands was performed by using Image J 1.46r (NIH, USA).

### Immunocytochemistry (ICC)

To confirm the effect of ASI on nuclear translocation of NF κB, ICC staining was conducted. BV-2 cells were pre-treated with or without ASI (50 μM) for 2 h prior to the stimulation of LPS (200 ng/ml). One hour later, the cells were washed with PBS twice followed by fixation with 4% PFA for 10 min. Afterwards, they were permeablized and blocked with 5% normal donkey serum in PBS containing 0.1% Triton X-100. Half an hour later, the cells were incubated with phospho-NF κB p65 antibody at 4 °C overnight. After thoroughly washed with PBS, they were further incubated with Alexa-594 conjugated secondary antibody at room temperature for 1 h. Thereafter, the cells were mounted with Vector mounting medium supplemented with DAPI after washed with PBS. The fluorescence of the cells was visualized under Olympus DX81 fluorescent microscope.

### Proinflammatory factors assay

BV-2 cells were pre-treated with or without ASI (50 μM) or Dex (10 μM) for 2 h and then co-stimulated with LPS (200 ng/ml) for 24 h. The culture medium was collected and subjected to the following assays. The concentration of TNFα was determined using a TNFα ELISA kit (eBiosciences, USA) by a TNFα standard curve prepared with recombinant protein. The amount of NO in the medium was determined with the Griess assay. Briefly, after incubated with nitrate reductase for 1 h at 37 °C to reduce nitrate to nitrite, the medium was mixed with equal volume of Griess reagent (Sigma). The optical density of the mixture was immediately measured at 540 nm with a Varioskan flash spectral scanning multimode reader (Thermo Scientific, USA). The concentration of nitrate in the medium was determined by a standard curve of sodium nitrate. Data obtained were expressed as fold change over controls.

### Luciferase activity assay

BV-2 cells were seeded in a 24-well plate at 2 × 10^5^ cells/well and cultured overnight. The cells were transiently co-transfected with NF κB reporter vector pGL6 (luc2P/NF-κB-RE/Hygro) (Beyotime, China) and Renilla luciferase plasmids using Lipofectamine™ LTX and Plus reagent (Life Technologies). Eighteen hours after transfection, the cells were treated with ASI (0, 10, 25, and 50 μM) for 2 h followed by stimulation with LPS (200 ng/ml) for an additional 24 h. At last, cells were washed once with PBS and lysed in 1 × passive lysis buffer (Promega, USA). The lysates were centrifuged at 10,000 rpm for 2 min at 4 °C. The luciferase activity of the supernatant was assayed using a Dual-luciferase Reporter assay system (Promega) with a Glomax 20/20 luminometer (Promega). To avoid the bias of transfection efficiency, final activities were designated as the activity of firefly luciferase normalized to that of Renilla luciferase.

HEK293 cells were seeded in a 24-well plate at 2 × 10^5^ cells/well and cultured overnight. Eighteen hours after being transiently transfected with GR reporter vector pGRE-luciferase (Beyotime) and Renilla luciferase plasmids, the cells were pre-treated with RU486 (1 μM) for 30 min followed by Dex (10 μM) or ASI (10, 25, and 50 μM) treatment. Thirty hours later, the cells were lysed and subjected to luciferase activity assays as described above. Concentration of Dex used was determined according to the previously published report^44^.

### Quantitative PCR

Total RNAs from the tissues or cells were extracted using Trizol reagent (Life Technologies). After eliminating trace amounts of DNA contamination with DNase I, total RNA was reversely transcripted into cDNA with Prime Script RT reagent (TakaRa, China). Quantitative PCR was carried out using Taqman SYBR kit (Life Technologies). Quantity of target genes was determined by standard curves generated with template plasmids containing fragments of respective target genes. Thereafter, they were normalized to that of glyceraldehydes-3-phosphate dehydrogenase (GAPDH) in the same sample. The sequences of forward and reverse primers were listed as follows: for iNOS, 5′-AACATCAGGTCGGCCATCAC-3′ and 5′-CCAGAGCCTGAAGTCATGTTTG-3′; for TNFα, 5′-AACCTCCTCTCTGCCGTCAAG-3′ and 5′-CCTCCCAGGTATATGGGCTCAT-3′; for IL1β, 5′-TGGGCCTCAAAGGAAAGAATC-3′ and 5′-GGTATTGCTTGGGATCCACACT-3′; for CD11b, 5′-GGTCGGCAAGCAACTGATTT-3′ and 5′-CAACTTGCATTATGGCATCCA-3′; for GAPDH, 5′-ATGTGTCCGTCGTGGATCTGA-3′ and 5′-ATGCCTGCTTCACCACCTTCT-3′.

### Molecular docking experiment

The canonical sequence of mouse glucocorticoid receptor (mGR, P06537) was retrieved from UniProt (http://www.uniprot.org). The 3D structure of mGR was downloaded from Protein Database (PDB) by mapping the UniProt ID to PDB with the ID mapping tools on UniProt website and subjected to MOE 2013.08 (MOE) for structural consensus and flexibility analysis. An mGR steroid binding domain complexed with dexamethasone, 3MNE, was chosen as the starting model for docking analysis. The bread loop (G553 to P559), missing atoms, hydrogen and partial charge issues of the protein was fixed within MMFF94x forcefield using Structure Preparation tool in MOE. The hydrogen network was optimized by using Protonate 3D. Since S608 was close to the ligand binding pocket, a back mutation from serine to phenylalanine, was carried out by using the Rotamer Explorer to build a wild-type mGR steroid binding domain (SBD) model. Finally, whole complex was minimized with fixed heavy atoms except the atoms within 4.5Å around the mutated F608. The processed complex structure was saved for further docking study. All the docking and MD simulation were performed on Dell T5500 workstation installed with Quadro K600 graphic card.

### GR competitive ligand-binding assay

To confirm if ASI could bind to GR, a LanthaScreen TR-FRET glucocorticoid receptor competitive binding assay kit was used (Invitrogen). Briefly, series dilutions of ASI (0, 10, 20, 40, 80, and 100 μM) were competed with Fluormone^TM^ GS1 Green for binding with terbium labeled GR-LBD on a 384-well plate. One hour later after incubation at room temperature, the fluorescence intensity was detected on a microplate reader (Excitation: 340 nm; Fluorescein emission: 520 nm; Terbium emission: 490 nm; Envision^TM^, PerkinElmer). The final data were shown by normalizing the signal of fluorescein to that of terbium.

### Statistical analysis

Difference among groups was analyzed by one-way ANOVA using Graphpad Prism 5 software with Dunnett’s multiple comparison test (La Jolla, CA, USA). Meanwhile, t-test was employed to compare the difference between two groups. Difference was considered significant when *p* < 0.05. All data were presented as mean ± S.E.M.

## Additional Information

**How to cite this article**: Liu, H.-S. *et al.* Astragaloside IV inhibits microglia activation via glucocorticoid receptor mediated signaling pathway. *Sci. Rep.* **6**, 19137; doi: 10.1038/srep19137 (2016).

## Supplementary Material

Supplementary Information
