# Supplementary material for: Astragaloside IV inhibits microglia activation via glucocorticoid receptor mediated signaling pathway
Source: Sci Rep. 2016 Jan 11;6:19137. doi: 10.1038/srep19137 (PMC4707476; doi:10.1038/srep19137)

**Title page**

**Title**

Astragaloside IV inhibits microglia activation via glucocorticoid receptor mediated signaling pathway

Hong-Shuai Liu^1, #^, Hai-Lian Shi^1, #^, Fei Huang^1^, Karin E. Peterson^2^, Hui Wu^1^, Yun-Yi Lan^1^, Bei-Bei Zhang^1^, Yi-Xin He^1^, Tyson Woods^2^, Min Du^3, *^, Xiao-Jun Wu^1, *^, Zheng-Tao Wang^1^

**Running title:** ASI deactivates microglia via GR

*^1^Shanghai Key Laboratory of Complex Prescription, The Ministry of Education (MOE) Key Laboratory for Standardization of Chinese Medicines, Institute of Chinese Materia Medica, Shanghai University of Traditional Chinese Medicine, Shanghai, 201203, China*

*^2^Laboratory of Persistent Viral Diseases, Rocky Mountain Laboratories, National Institute of Allergy and Infectious Disease, Hamilton, Montana, 59840, USA*

*^3^Unit of Immune Signaling and Regulation, Key Laboratory of Molecular Virology and Immunology, Institut Pasteur of Shanghai, Chinese Academy of Sciences, Shanghai, 200030, China*

**Corresponding authors** ^*^

Xiaojun Wu, Institute of Chinese Materia Medica, Shanghai University of Traditional Chinese Medicine, 1200 Cailun Road, Shanghai, 201203, China. E-mail*:* [xiaojunwu320@126.com](mailto:xiaojunwu320@126.com); Tel.: +86 21 51322578; Fax: +86 21 51322505.

Min Du, Unit of Immune Signaling and Regulation, Key Laboratory of Molecular Virology and Immunology, Institut Pasteur of Shanghai, Chinese Academy of Sciences, Shanghai, 200030, China. E-mail*:* [mindu@ips.ac.cn](mailto:mindu@ips.ac.cn); Tel.: +86 21 54923126.

^#^These authors contributed equally to this work.

Figure 1B iNOS


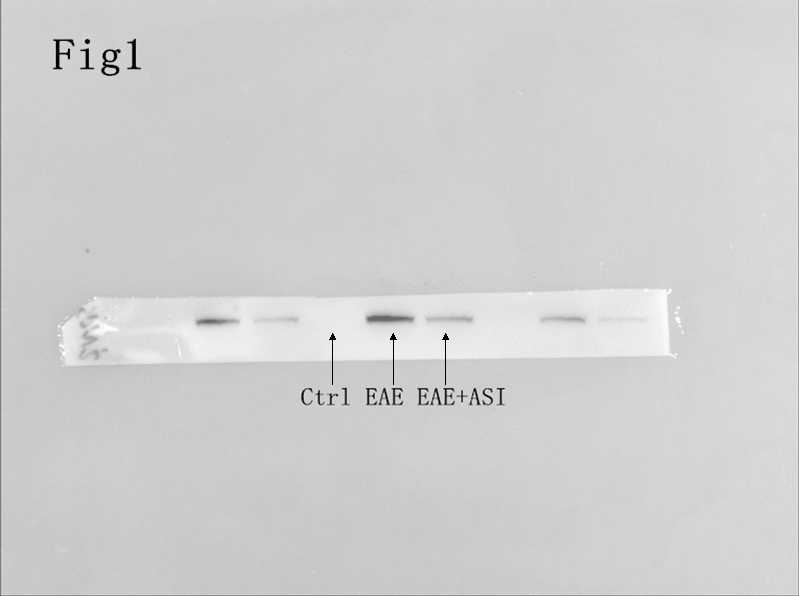


Figure 1B beta-actin


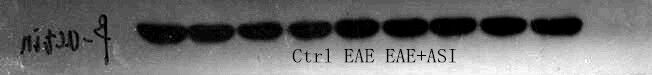


Figure 2B pp65 NF-κB


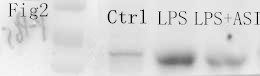


Figure 2B p65 NF-κB


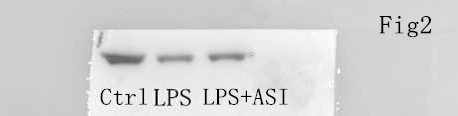


Figure 2B pI κB


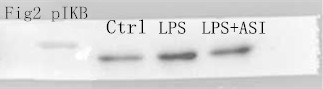


Figure 2B I κB


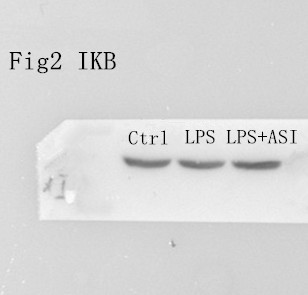


Figure 3E Iba-1


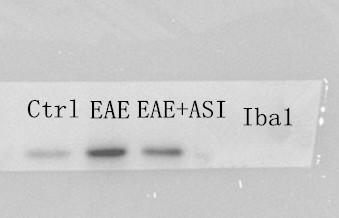


Figure 3E iNOS


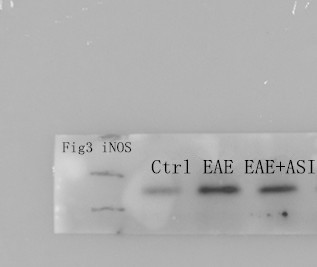


Figure 3E p-Tau


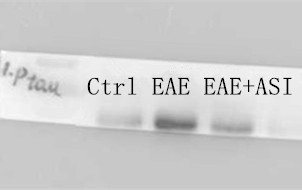


Figure 3E Tau


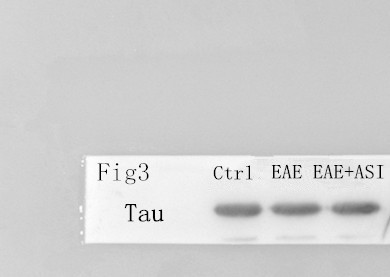


Figure 3E beta-actin


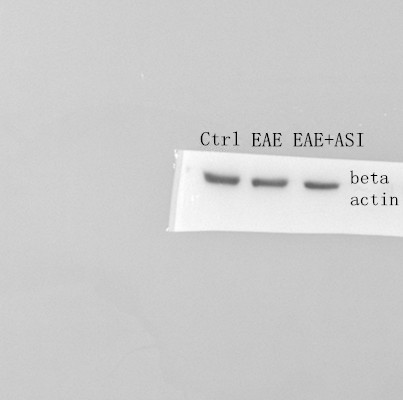


Figure 4B GR


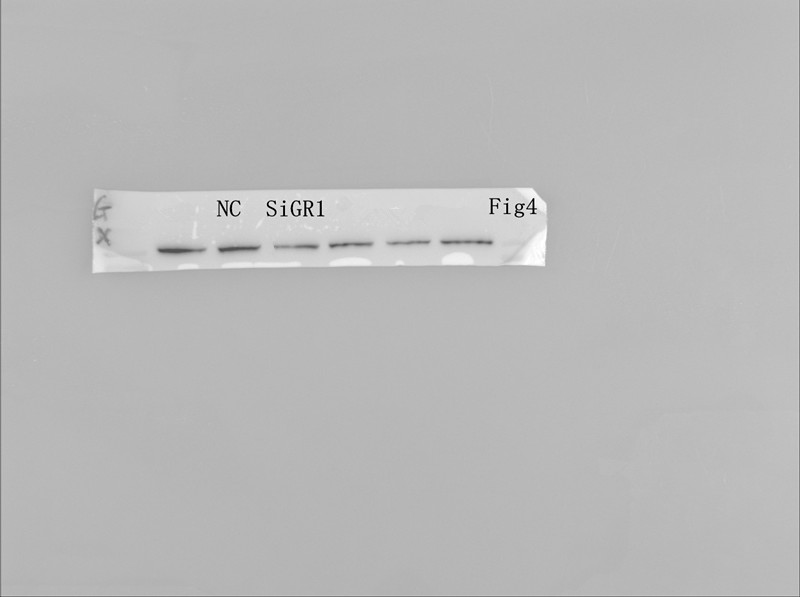


Figure 4B β-actin


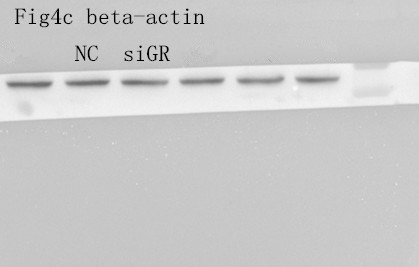


Figure 4C iNOS


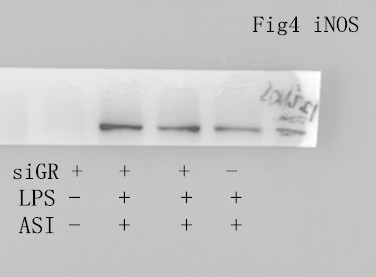


Figure 4C β-actin


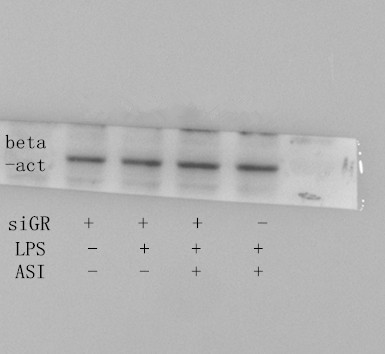


Figure 4E


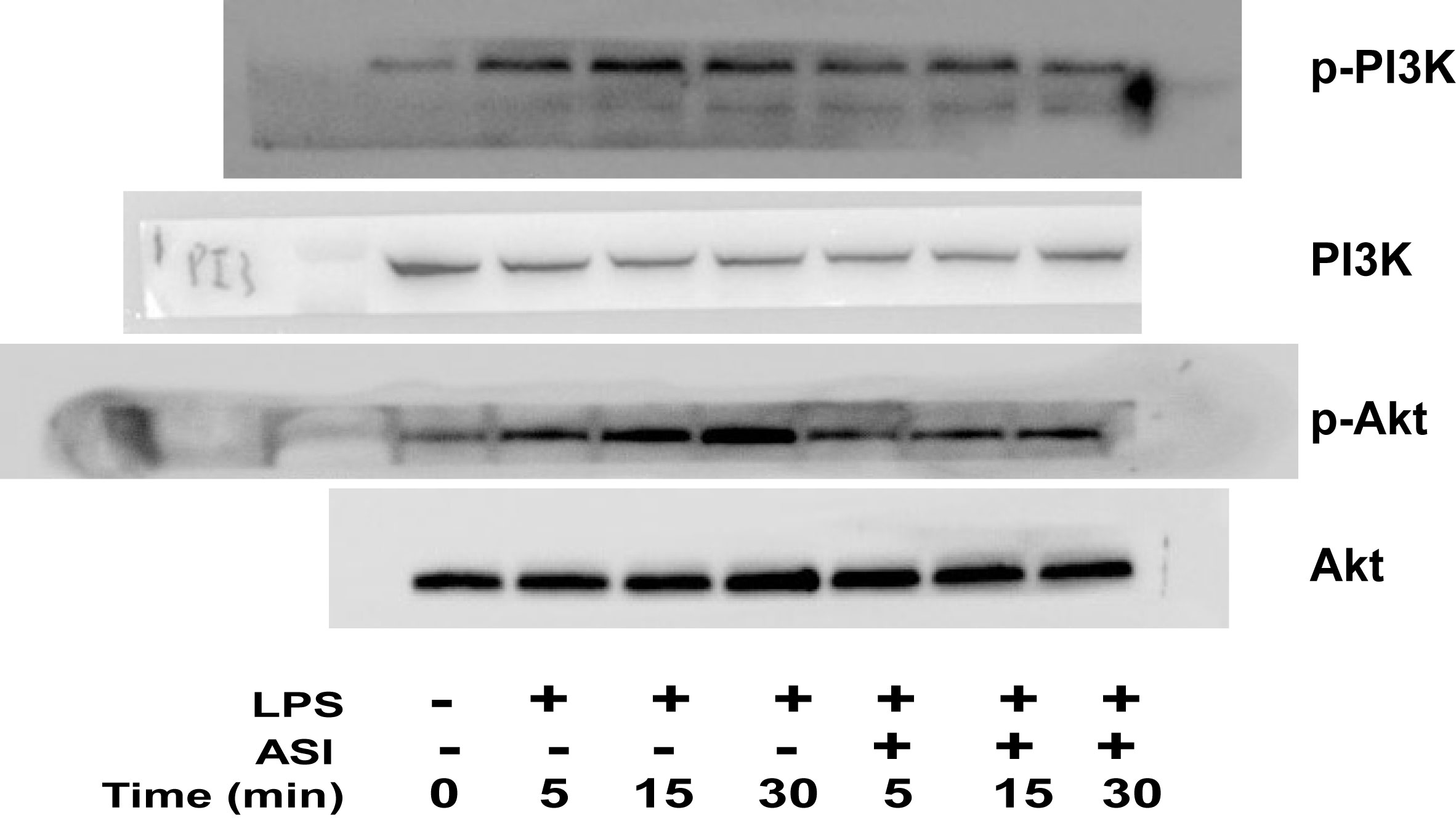


Figure 4F


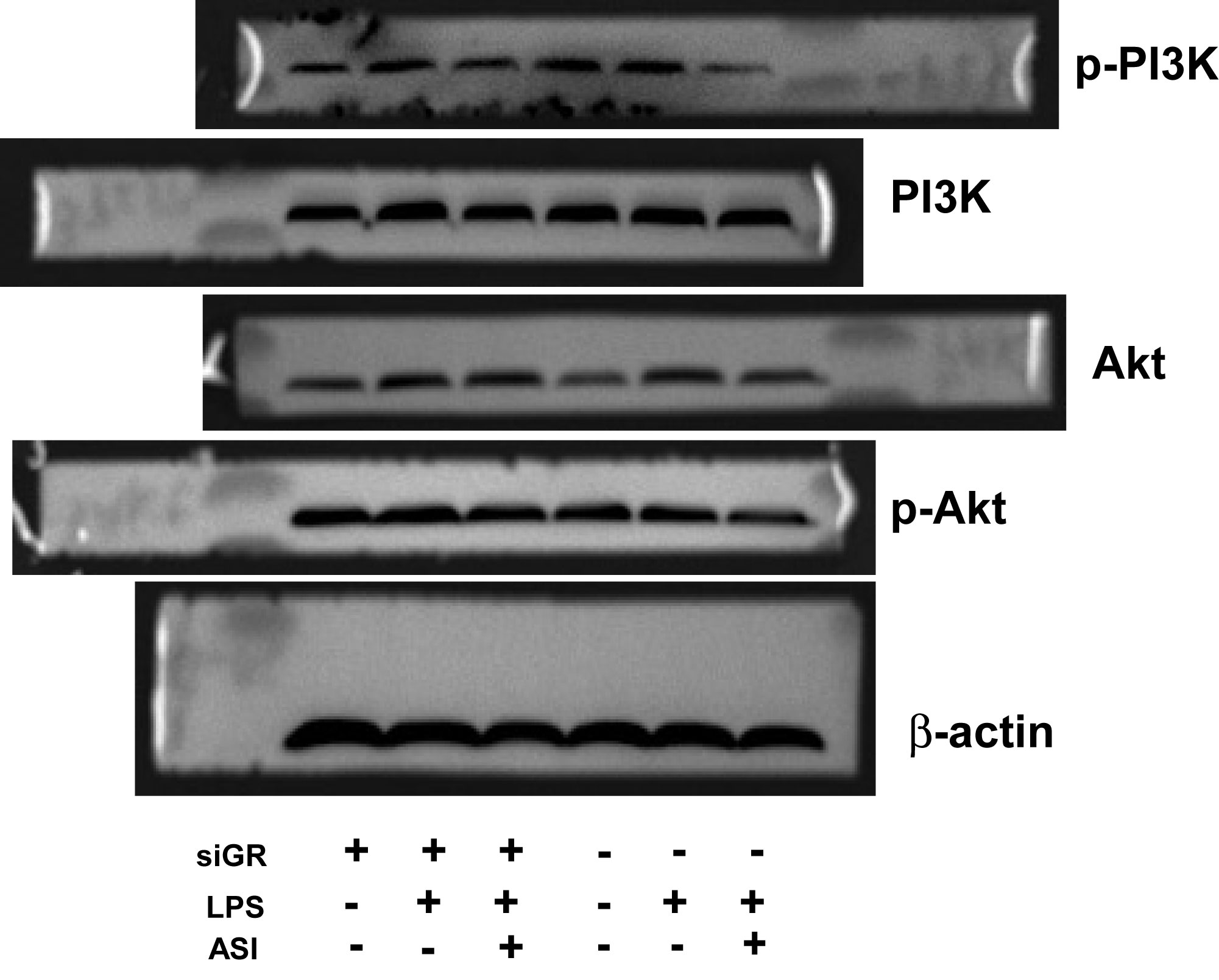


Figure 5B


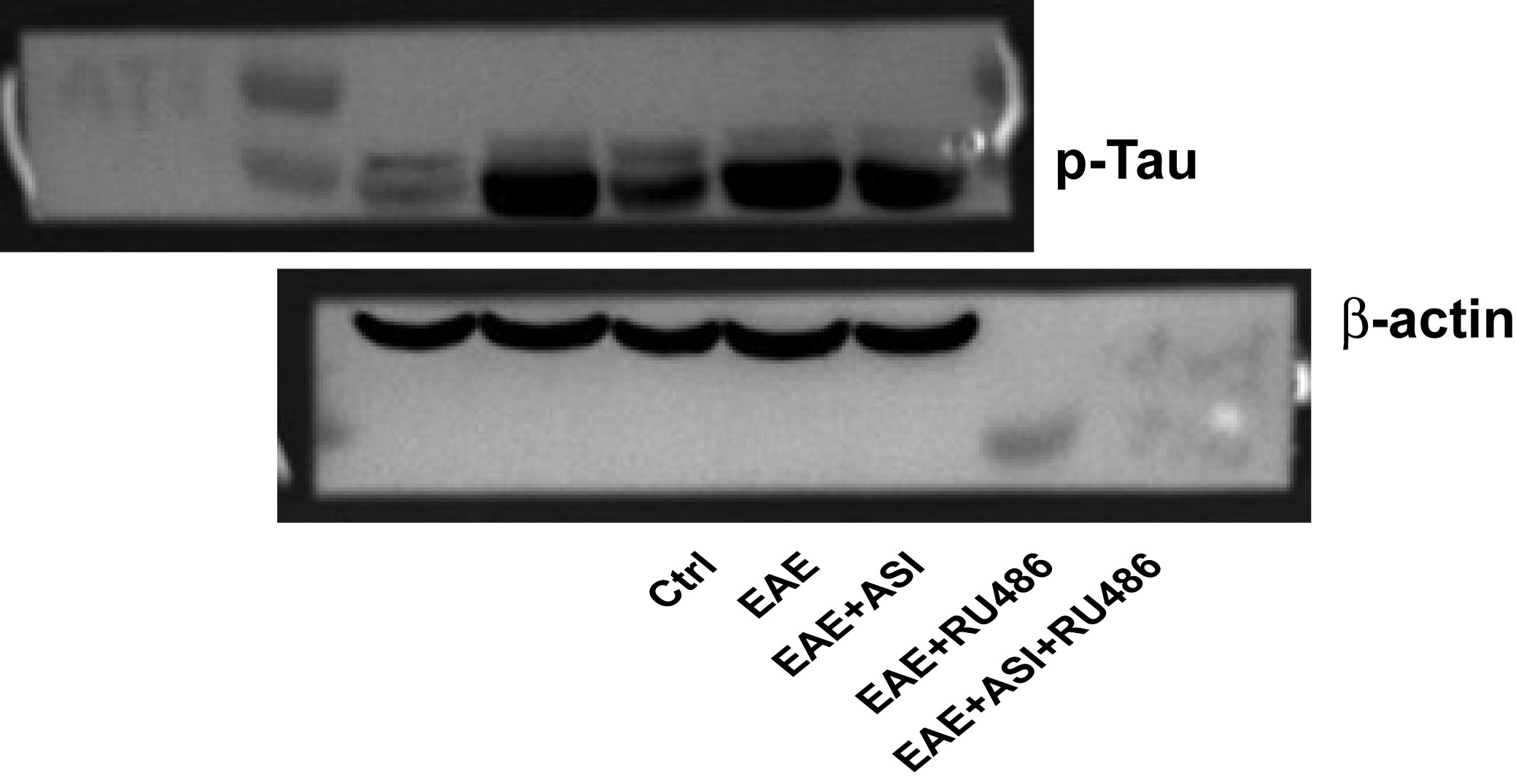


Figure 6B


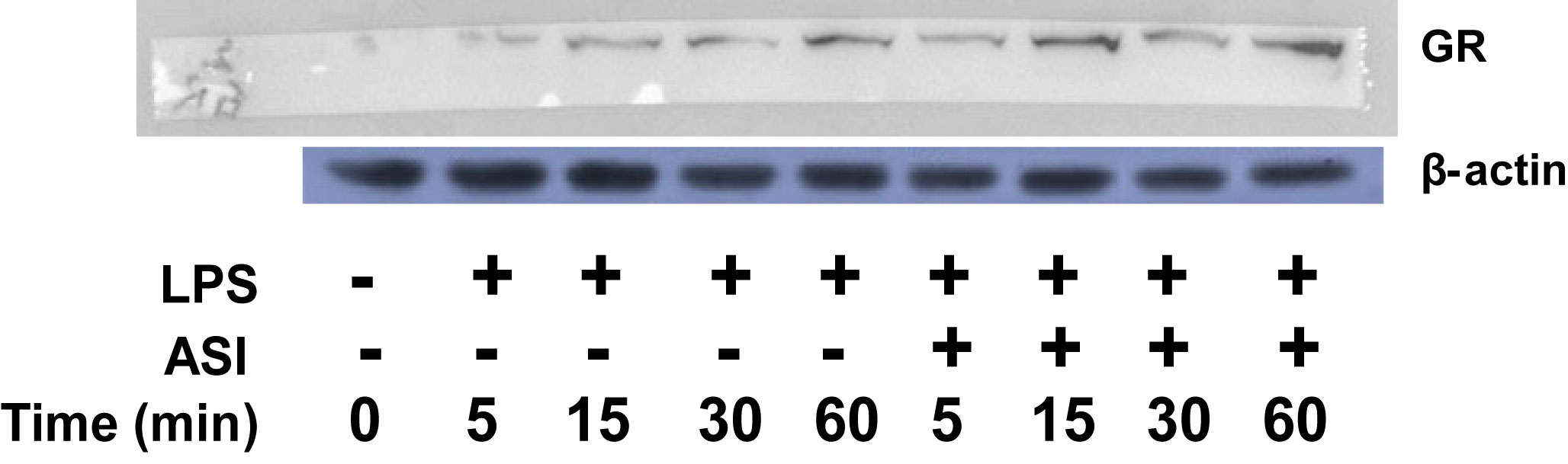

Supplement: Supplementary Information [file srep19137-s1.doc]
